# FRET-based Tau seeding assay does not represent prion-like templated assembly of Tau filaments

**DOI:** 10.1186/s13024-020-00389-1

**Published:** 2020-07-16

**Authors:** Senthilvelrajan Kaniyappan, Katharina Tepper, Jacek Biernat, Ram Reddy Chandupatla, Sabrina Hübschmann, Stephan Irsen, Sandra Bicher, Christoph Klatt, Eva-Maria Mandelkow, Eckhard Mandelkow

**Affiliations:** 1 DZNE, German Center for Neurodegenerative Diseases, Bonn, Germany; 2 Department of Neurodegenerative Diseases and Geriatric Psychiatry, University of Bonn, Bonn, Germany; 3 CAESAR Research Center, Bonn, Germany

**Keywords:** Tau protein, Propagation, Seeding, Alzheimer, Amyloid

## Abstract

Tau aggregation into amyloid fibers based on the cross-beta structure is a hallmark of several Tauopathies, including Alzheimer Disease (AD). Trans-cellular propagation of Tau with pathological conformation has been suggested as a key disease mechanism. This is thought to cause the spreading of Tau pathology in AD by templated conversion of naive Tau in recipient cells into a pathological state, followed by assembly of pathological Tau fibers, similar to the mechanism of nucleated polymerization proposed for prion pathogenesis. In cell cultures, the process is often monitored by a FRET assay where the recipient cell expresses the Tau repeat domain (Tau^RD^) with a pro-aggregant mutation, fused to GFP-based FRET pairs. Since the size of the reporter GFP (barrel of ~ 3 nm × 4 nm) is ~ 7 times larger than the β-strand distance (0.47 nm), this points to a potential steric clash. Hence, we investigated the influence of the GFP tag on Tau^FL^ or Tau^RD^ aggregation. Using biophysical methods (light scattering, atomic force microscopy (AFM), and scanning-transmission electron microscopy (STEM)), we found that the assembly of Tau^RD^-GFP was severely inhibited and incompatible with that of Alzheimer filaments. These observations argue against the hypothesis that the propagation of Tau pathology in AD is caused by the prion-like templated aggregation of Tau protein, transmitted via cell-to-cell spreading of Tau. Thus, even though the observed local increase of FRET in recipient cells may be a valid hallmark of a pathological reaction, our data argue that it is caused by a process distinct from assembly of Tau^RD^ filaments.

## Background

Tau, a microtubule-associated protein (MAPT, Uniprot P10636), has an important role in microtubule assembly and stabilization. Tau has a hydrophilic, mostly basic composition, is natively unfolded and is highly soluble. Nevertheless, Tau amyloidogenic aggregates characterize a wide range of neurodegenerative diseases known as Tauopathies [35, 45, 73] including Alzheimer Disease (AD). Mutations in the Tau gene alone are sufficient to cause neurodegeneration [36]. Moreover Tau deposits in the brain correlate well with the memory decline, confirming the importance of Tau pathology in AD (Braak stages) [13, 54]. Biophysical and structural studies show that soluble monomeric Tau, upon nucleation by polyanionic cofactors like heparin or RNA, can form insoluble paired helical filaments (PHFs) in vitro [24, 38]. However, the pathways causing Tau aggregation in neurons and of Tau-induced neurodegeneration are not well understood.

In AD, Tau pathology spreads from the entorhinal cortex to anatomically connected regions such as hippocampus, subiculum and cortex. The spatio-temporal progression of cognitive impairment correlates well with the Tau pathology, as assessed by hallmarks such as aggregation or hyperphosphorylation [12, 13]. This has led to the hypothesis that the disease progression in AD is caused by the cell-to-cell spreading of Tau protein itself in a pathological state [34, 41], rather than by some other signal. Consistent with this hypothesis, Tau can be secreted from neurons (concentration in ISF ~ 1 nM [81]), secretion is enhanced by neuronal activation [61, 80], by exosomes [72] and by neuronal death [5]. Extracellular Tau can be taken up by neighboring cells by several mechanisms including receptor mediated endocytosis, phagocytosis, muscarinic receptor-mediated or HSPG mediated uptake [26, 29, 32, 49]. The internalized Tau is thought to induce the fibrous aggregation of endogenous Tau by templated self-assembly. This would promote further aggregation and propagation of Tau pathology to other cells, by analogy to the mechanism proposed for prion pathogenesis [18, 62] which is based on the concept of nucleated protein polymerization [60]. Following the assumption that spreading of Tau protein is responsible for the spreading of neuronal pathology, current therapeutic approaches include the prevention of the pathological conformation of Tau, scavenging extracellular Tau by antibodies, blocking of Tau uptake by neurons, reducing Tau concentrations, and others [20, 66, 82].

The key methods for investigating the reactions of cellular Tau protein in response to external Tau are based on expressing aggregation-prone forms of Tau repeat domains (Tau^RD^) labeled with fluorescent sensors, e.g. CFP, YFP. Their accumulation can be observed by local increases of fluorescence, FRET, or FLIM which indicate proximity within several nm [27, 41]. The repeat domain (RD) of Tau represents the assembly-competent core of Tau filaments, as it contains the hexapeptide motifs with a high propensity to generate β-structure [70, 79]. Mutations such as ΔK280, P301L, P301S in the repeat domain enhance β-propensity of Tau and hence show stronger toxic effects [51, 63]. Although a FRET signal in a recipient cell requires only proximity of the XFP labels (< 10 nm) it is usually taken as a sign of pathological PHF-like Tau filaments, induced by the pathological conformation of the Tau subunits (termed seeds) penetrating into the cells. This would amount to a prion-like propagation of Tau pathology from cell to cell [34].

**Fig. 1:**
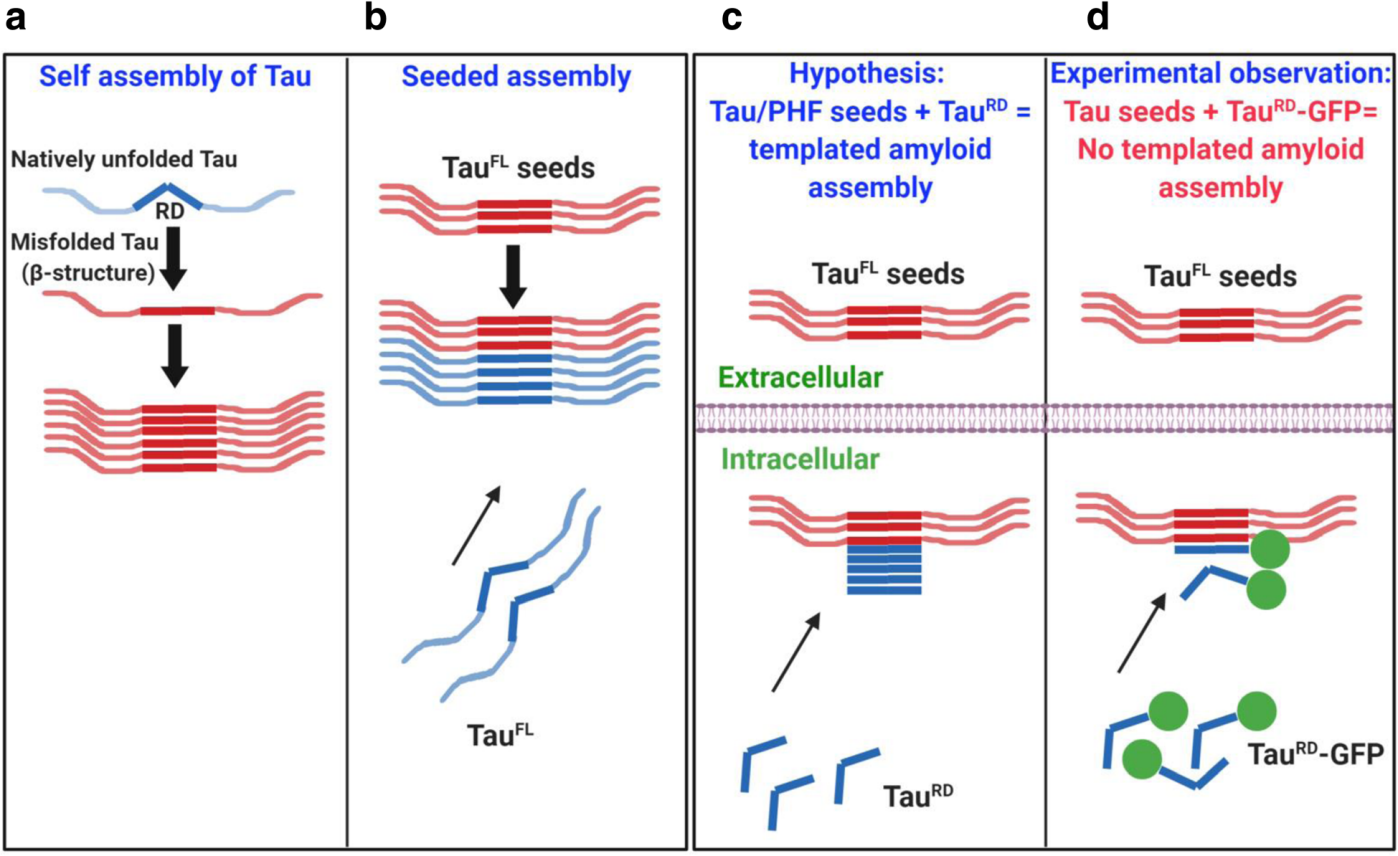
Schematic representation of effects of GFP label in preventing templated assembly of Tau^RD^. **(a)** Self-assembly of Tau: Tau protein is highly hydrophilic and natively unfolded. In disease conditions, Tau becomes misfolded, attains β-structure and assembles into paired helical filaments. **(b)** Seeded (templated) assembly: Tau protein subunits elongate into long filaments using external Tau seeds as template (usually prepared by sonication of preformed fibrils). Note: In Tau assembly experiments in vitro, polyanions such as heparin must be added to initiate nucleation. **(c)**Pre-dominant hypothesis: In cell culture, Tau seeds (made from Tau^FL^ or Tau^RD^) are internalized into acceptor cells. Endogenous Tau^RD^ is thought to attain the amyloid structure of the seeds and elongate the filaments. **(d)** Experimental observation: In cell culture studies Tau^RD^-GFP is often used as endogenous Tau fusion protein, intended to elongate templating seed structures. However, steric hindrance by the large GFP molecule prevents fiber formation

From a structural perspective, the templated assembly of Tau into bona fide PHFs can only be concluded if the Tau aggregates are filaments with cross-β structure (axial repeat between strands ~ 0.47 nm), similar to PHFs from AD brain [22]. We hypothesized that a large GFP reporter molecule (~ 28 kD) tagged onto Tau^RD^ (~ 13 kD) could inhibit the aggregation because of a steric clash: The size of the reporter GFP (a barrel-shaped molecule ~ 3 × 4 nm) [83] is ~ 7 times larger than the β-strand distance (0.47 nm) between the Tau molecules (Fig. 1). To test this, we investigated the assembly forms of Tau^RD^ tagged with GFP either at their C-terminus or N-terminus, using several biophysical and microscopic methods. For comparison we also studied the assembly forms of GFP-tagged full-length Tau, and the same Tau proteins without GFP tags. The results showed that the self-assembly of Tau^RD^ is severely inhibited when tagged with GFP. In particular, even when fiber-like particles occurred they had a very different structure and mass distribution, distinct from that of PHF-like Tau fibers. We conclude that cell inclusions with enhanced FRET intensity do not result from a templated assembly of PHF-like Tau filaments, and do not result from the transfer of a pathological conformation.

## Materials and methods

### Materials

All chemicals were obtained from Sigma-Aldrich (Taufkirchen, Germany), Fluka (Seelze, Germany), and Roth (Karlsruhe, Germany) in highest purity if not stated otherwise. Heparin 5000 or 16,000 was purchased from Fisher Scientific (#BP2524–10). MSNL-10 probe for AFM measurements were from Bruker, USA.

### Cells and viruses

Sf9 cells were obtained from Invitrogen (Life Technologies, DE) and grown at 27 °C in monolayer culture with Grace’s medium (Sigma /Life Technologies TM) supplemented with 10% fetal bovine serum and 1% penicillin / streptomycin (PS).

Sapphire Baculovirus DNA was obtained from Orbigen/Biozol (Eching, DE) and pVL1392 from Invitrogen (Life Technologies, EM). MultiBacTurbo system contains the acceptor plasmid pACEBac1 and the E.coli strain DH10MultiBac /Turbo contains the acceptor bacmid. MultiBacTurbo baculoviral DNA were obtained from EMBL Grenoble Outstation [10].

### Plasmids and baculovirus construction

The hTau40 cDNA, the longest Tau isoform in human CNS (2N4R), was tagged with 6xHis at the C-terminus using PCR amplification and the modified Tau-His cassette was introduced into pET3a plasmid (Novagen, DE) linearized with NdeI enzyme succeeding in pET3a/htau40/6xHis plasmid for the protein expression in E.coli. The cDNA cassette of GFP-hTau40 (consisting of M1 – L239 of GFP sequence bridged by one aa: His to htau40 (M1 – L441) sequence elongated with 6x His at the C-terminus was inserted into pET3a plasmid succeeding in E.coli expression vector pET3a GFP/htau40/6xHis.

The cDNA cassette of htau40-GFP (consisting of htau40 (M1-L441) bridged by 13 aa AH linker sequence: GAPGSAGSAAGSG to M1- L239 GFP terminated with 6xHis Tag was also inserted into pET3a plasmid leading to the pET3a htau40/GFP/6xHis expression vector for E.coli. Similarly, the cDNA cassette of GFP-Tau^RDΔK^-6xHis consisting of GFP, pro-aggregant Tau repeat domain (4-repeat construct K18, M-Q244-E372, with the deletion mutation ΔK280 terminated with 6xHis Tag (GFP-Tau^RDΔK^) was also inserted into pET3a plasmid, yielding the pET3a GFP-Tau^RDΔK^-6xHis expression vector. This mutation strongly accelerates aggregation due to pronounced β propensity [68].

A second group of proteins: Tau^RDΔK^ –GFP and GFP/6xHis proteins was expressed in the baculovirus expression system. A cDNA cassette containing Tau^RDΔK^ tagged with GFP at its C-terminus (Tau^RDΔK^–GFP) followed by a 6xHis tail was inserted into pVL1392 vector resulting in the plasmid pVL1392 Tau^RDΔK^/GFP/6xHis. After mixing with Saphire TM baculovirus DNA, this plasmid was used for the generation of baculovirus and protein expression in Sf9 insect cells as described before [11].

The GFP cDNA sequence fused to a 6xHis tail at its C-terminus was inserted into the pACEBac-1 acceptor vector to generate the pACEBac/GFP/6xHis plasmid. This plasmid was subsequently subjected for the Tn7- dependent integration into the baculoviral genome of DH10 MultiBacTurbo E.coli cells for the generation of baculovirus encoding GFP-His-tagged protein. The purified MultiBacTurbo bacmid encoding GFP protein was transfected into Sf9 cells for the generation of baculoviruses and subsequent protein expression [10].

**Fig. 2:**
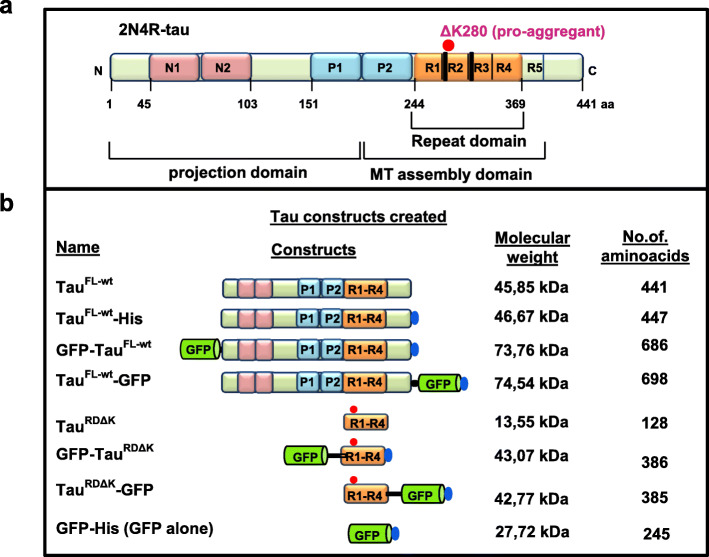
Tau proteins and variants used in this study. **(a)** Bar diagram of longest isoform of Tau in human CNS comprising 441 amino acids (Uniprot P10636-F, alias hTau40, Tau-2N4R). Domains are depicted with 2 near-N-terminal inserts (N1, N2, pink), the proline-rich domains (P1, P2; blue), 4 pseudo-repeats (R1-R4, ~ 31 residues each, ochre, plus one less-conserved repeat R5), and the C-terminal tail. The amyloidogenic hexapeptide motifs are indicated at the beginning of R2, R3 (thick black line). The C-terminal half (MT assembly domain P2-R5) promotes both microtubule assembly and pathological aggregation of Tau, the N-terminal half (projection domain) projects from the surface of microtubules or from the core of Tau fibers, respectively. The “pro-aggregant” deletion mutation ΔK280 (red dot) lies near the beginning of R2, it increases the β-propensity of Tau and hence increases pathological aggregation. **(b)** Tau and GFP fusion proteins are schematically presented with their name listed on the left. The GFP-tag is positioned either at the N-terminus or C-terminus. Some constructs contain a C-terminal 6xHis-tag to aid in the purification (blue ellipse). A sequence of 13–14 amino acids was used as a linker (black line) in the two short Tau constructs GFP-Tau^RDΔK^ and Tau^RDΔK^-GFP to increase the distance between the GFP and the repeat domain (see Table S1)

An overview of the Tau constructs is shown in Fig. 2 (for sequence see Supplement Table S1).

### Protein preparation and purification

Proteins were expressed either in E.coli or in insect Sf9-cells using the baculovirus expression system. The Tau proteins hTau40wt (2N4R) and Tau^RDΔK^ (K18 construct, (M) Q244-E372 with deletion of K280) were expressed in E.coli and purified as described earlier [8].

The proteins Tau-His (hTau40/6xHis; 2N4R), GFP-Tau^RDΔK^ (GFP- (M)Q244-E372 with deleted K280/6xHis) and GFP-Tau (GFP- hTau40/6xHis; 2N4R) and Tau-GFP (hTau40/−GFP/6xHis; 2N4R) were expressed as fusion proteins with 6x polyHis tail at the C-terminus in E.coli strain BL21(DE3) (Merck-Novagen, Darmstadt). After harvesting, the E.coli bacteria cell pellet was directly re-suspended in lysis buffer [50 mM Tris HCl pH 7.3, 300 mM NaCl, 10% glycerol, 0.5 mM TCEP, 10 mM Imidazol, 1 mM Benzamidin, 1 mM PMSF and 10 μg/ml each of protease inhibitors leupeptin, aprotinin, and pepstatin], in the ratio 1 g E.coli pellet to 10 ml lysis buffer and disrupted by French press.

Protein Tau^RDΔK^-GFP (M) Q244-E372 with deletion of K280-GFP/6xHis), GFP/6xHis and also GFP-Tau (GFP- hTau40/6xHis; 2N4R) and (hTau40/−GFP/6xHis; 2N4R) were expressed in Sf9 insect cells from Invitrogen. Sf9 cells were infected with recombinant virus at a MOI of > 1 typically in six T150 cell culture flasks containing 75% confluent Sf9 cells. Cells were incubated for 3 days at 27 °C, collected and re-suspended for preparation in the lysis buffer [50 mM Tris HCl pH 7.3, 300 mM NaCl, 10% glycerol, 0.5 mM TCEP, 10 mM Imidazol, 1 mM Benzamidin, 1 mM PMSF and 10 μg/ml each of protease inhibitors leupeptin, aprotinin, and pepstatin], in the ratio 1 g Sf9 pellet to 10 ml lysis buffer and disrupted by French press.

The lysates of the E.coli bacteria and Sf9 cells were cleared by centrifugation in a Beckman Optima L- 80 XP Ultracentrifuge with a Ti45 rotor (40,000 rpm, for 60 min at 4 °C), applied to Ni^2+^ ion affinity chromatography His Trap FF column (GE Healthcare), and purified using an Äkta pure chromatography system (GE Healthcare). Following extensive wash with 12 column volumes (CV) of wash buffer (50 mM Na phosphate buffer pH 7.2, 300 mM NaCl, and 25 mM imidazole) the protein was eluted with elution buffer (50 mM Na Phosphate buffer pH 7.2, 300 mM NaCl and 1 M imidazol).

If necessary the protein breakdown products were separated in the second chromatography step using gel filtration on a Superdex G200 column (GE Healthcare, Freiburg). PBS buffer was used for gel filtration column (137 mM NaCl, 3 mM KCl, 10 mM Na_2_HPO_4_, 2 mM KH_2_PO_4_, pH 7.4) with 1 mM DTT (freshly added).

### Polymerization assays

#### Light scattering

Tau aggregation was monitored by 90° angle light scattering at 350 nm in a FluoroMax spectrophotometer (HORIBA). 50 μM Tau protein is suspended in BES buffer, pH 7.0 (20 mM BES, 25 mM NaCl) and supplemented with 12.5 μM heparin 16,000 or heparin 5000. Heparin 5000 and 16,000 induce the aggregation at the same level and there is no difference between the aggregates formed under these conditions. This mixture was incubated at 37 °C. At different time points 20 μl of the samples were analyzed by light scattering at 350 nm and then the samples were brought back to original tube for further aggregation.

#### Sedimentation assay and western blotting

The aggregated Tau samples (t = 48 h) were sedimented at 61,000 rpm (TLA 100.3 rotor) for 1 h at 4 °C. The supernatant was collected and the pellet was resuspended to the same volume as supernatant. 5 μl of the samples were resolved on 10% SDS gel and immunoblotted as described in [40]. Later the blot was probed with K9JA (1:5000 dilution) and GFP (1:1000 dilution) antibodies and the signal was detected by chemiluminescence method.

#### Turbidity assays for MT assembly

Tau-induced microtubule assembly was monitored by 90° angle light scattering at 350 nm in a FluoroMax spectrophotometer (HORIBA). 10 μM PC-purified Tubulin were mixed with 5 μM Tau protein in RB-Buffer (100 mM PIPES pH 6.9, 1 mM DTT, 1 mM MgSO_4_, 1 mM EGTA, 1 mM GTP). The polymerization was started by transferring the ice-cold Tubulin-Tau-solution to the 37 °C warm cuvette-holder and the reaction started once the temperature was reached. Tubulin assembly was monitored for 15 min.

### Electron microscopy and mass per length analysis

#### Sample preparation

Holey carbon grids (Quantifoil R2/1) covered with either 2 nm amorphous carbon (Quantifoil, R2/1 + 2 nm C) or graphene were used for sample preparation. The preparation of graphene grids was done according to a method described earlier [57]. The sample concentration was adjusted for MPL measurements to a final concentration of 5–10 molecules / grid hole. The protein solution was mixed with Tobacco mosaic virus (TMV) prior to application to the grids. The TMV was used to calibrate the mass measurements. 10 μl sample was applied to the grids for 2 min. Excess liquid was blotted away using filter paper. Samples were washed 3 times with doubled distilled water to remove buffer salts and afterwards air dried or frozen and vacuum dried at − 80 °C for 12 h.

#### Scanning-transmission electron microscopy (STEM)

For the MPL experiments, a Zeiss Libra200 MC Cs-STEM CRISP (Corrected Illumination Scanning Probe) was used. The instrument was operated at 200 kV. The CRISP is equipped with a monochromated Schottky-type field emission cathode and a double hexapole-design corrector for spherical aberrations of the illumination system (Cs-corrector). A high-angle annular dark field (HAADF) detector (Fischione Instruments, USA) was used for imaging. The images were recorded at a convergence angle of 16 mrad and an acceptance angle of 20 mrad. Images were recorded at a pixel size of 0.6 nm with a dwell time of 70 μs/px. The total dose per image was 400 e/nm^2^.

Mass determination was done using the software PCMASS32 [64]. In a first step images were calibrated using tobacco mosaic virus as a calibration sample. Filament regions were manually selected and masked. The full datasets were plotted as histograms and fitted to Gaussian curves.

#### Transmission electron microscopy (TEM)

Samples for negative stain transmission electron microscopy were placed on 200 mesh formvar-carbonated copper grids with (Plano, Quantifoil #S162). Grids were glow-discharged for 30 s (Baltec, MED010) prior to sample addition. The protein solution was removed after 3 min incubation time by filter-paper and the grids washed 3 times with ddH_2_O (grid on top of each drop) to remove buffer salts, then followed by staining with 2% uranyl acetate for 60 s and finally removing the stain slowly with a wet-filter paper and rapid air drying. To prepare grids with microtubules for TEM, all steps were carried at out at 37 °C and with pre-warmed solutions. All specimens were analyzed at 200 kV using a JEOL JEM-2200FS TEM.

### Atomic force microscopy (AFM)

AFM measurements were performed as described earlier [39]. Briefly, Tau fibril samples were diluted in PBS buffer for a final concentration of 0.5 – 1 μM. 30 μl of the sample was placed onto freshly cleaved mica and allowed to adsorb for 10 min at room temperature. Unattached and excess proteins were removed by rinsing the sample with PBS for 4–5 times. Finally, the sample on the mica was filled with imaging buffer (10 mM Tris-HCl, pH 7.4, 50 mM KCl). AFM imaging was performed in an oscillation mode using JPK NanoWizard® ULTRA Speed AFM system and MSNL-10 probe with “F” cantilever. The amplitude set point and the gains were adjusted manually to control the thermal drift and to achieve the minimal force between the cantilever and the sample. AFM images were processed by JPK data processing software. Fibril heights/widths were measured by the inbuilt cross-sections method of JPK data processing software. For the analysis of fibril width 50 fibrils per condition were measured.

## Results

### Generation of Tau constructs to study the effect of fusion protein (GFP) on Tau.

For in vitro studies, GFP-tags were fused to either full length Tau protein (2N4R) or the shorter repeat domain containing the “pro-aggregant” deletion mutation ΔK280 (Tau^RDΔK^) at their N-terminus or C-terminus using a flexible linker (Fig. 2 a,b). The proteins were expressed in *E. coli*, except Tau^RDΔK^-GFP which could only be expressed in the baculovirus-Sf9 cell system. His-tags were added at the C-termini to aid in purification.

### GFP does not interfere with microtubule assembly induced by full-length Tau.

**Fig. 3:**
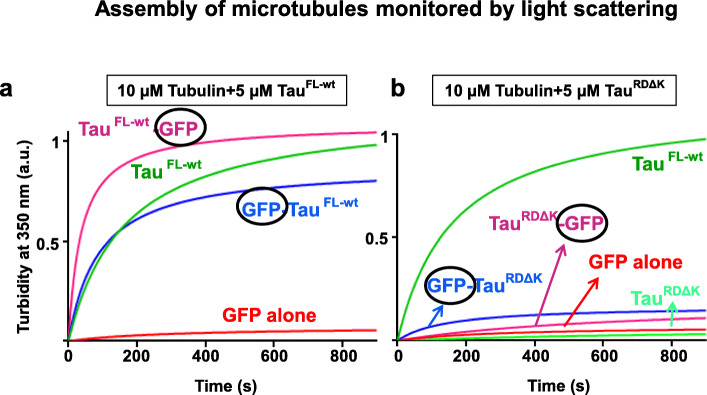
Full length wildtype Tau proteins (with or without GFP) induce microtubule assembly whereas the repeat domain does not. **(a)** Assembly of microtubules (10 μM tubulin) is supported by Tau^FL-wt^ (5 μM) without GFP or with GFP attached to either N- or C-terminus (top, green, blue, magenta curves). MT assembly levels and rates (t_1/2_ ~ min range) are comparable. Note that GFP alone shows no increase in light scattering (red curve). **(b)** Microtubule assembly is not supported by the repeat domain Tau^RDΔK^, regardless of GFP (bottom curves: Tau^RDΔK^ (light green); GFP-Tau^RDΔK^ (blue); Tau^RDΔK^–GFP (magenta); GFP alone (red)). As a control, Tau^FL-wt^ induces robust microtubule assembly (as in **a**, green). Note that the repeat domain R1-R4 is often denoted as “MTBD” domain in the literature, even though it binds and assembles microtubules poorly

We first asked whether GFP fusion to Tau affects the physiological function of promoting microtubule assembly as monitored by light scattering at 350 nm [23] and verified by electron and fluorescence microscopy (Fig. S1A-C). Full length Tau, either untagged or labeled with GFP at either end, is competent to induce MT assembly with similar time courses (t_1/2_ ~ 1-2 min, Fig. 3a, top curves). GFP alone does not support MT assembly (Fig. 3a, bottom curve). By contrast, the Tau repeat domain alone, either with or without ΔK280 mutation, and with or without GFP tag, is not competent to cause MT polymerization (Fig. 3b, bottom curves). This is consistent with the weak binding of Tau^RD^ to tubulin and microtubules, which becomes pronounced only when the domains flanking the repeats are present [30]. The results mean that GFP moieties attached to the either end of full-length Tau are sufficiently flexible and/or far from the MT binding domain to allow assembly without steric hindrance.

### GFP tags create steric hindrance against aggregation of Tau repeat domain

**Fig. 4:**
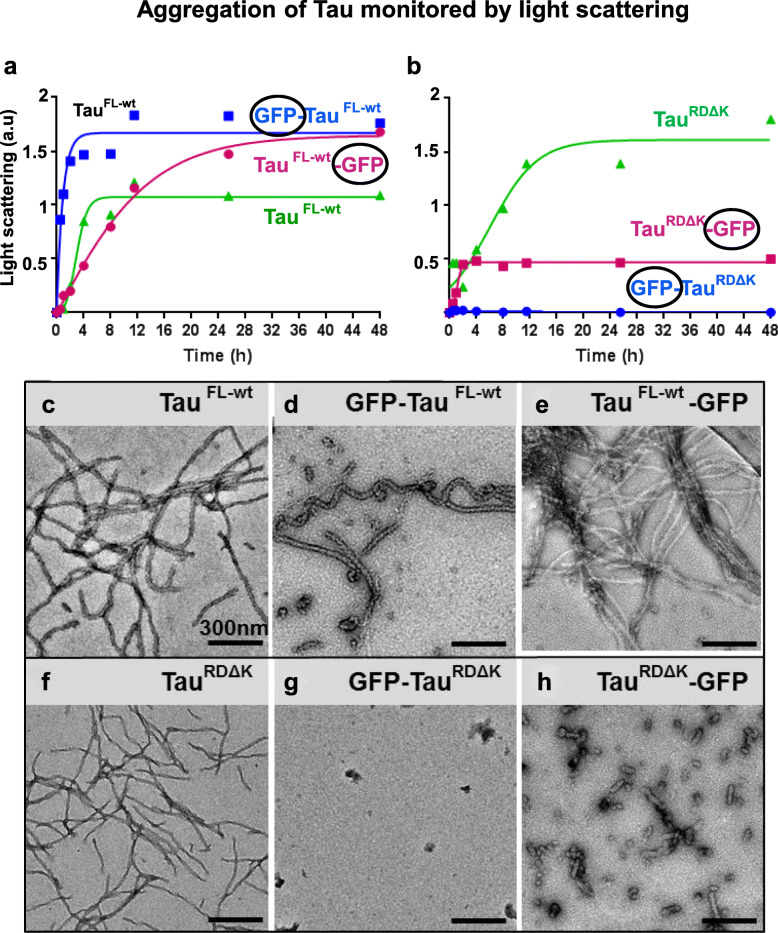
Aggregation of Tau proteins with GFP tags monitored by light scattering and electron microscopy. (**a**-**b**) Attachment of GFP to Tau repeat domain severely inhibits aggregation, but full length Tau+GFP remains aggregation competent. Aggregation of Tau to PHF-like fibers was monitored by light scattering at 350 nm. 50 μM of Tau^FL-wt^ or Tau^RDΔK^ with or without GFP on either end was incubated in the presence of heparin 16,000 (12.5 μM) in BES buffer, pH 7.0, at 37 °C at different time intervals. **(a)** Unlabeled Tau^FL-wt^ (green) aggregates mostly into PHF-like fibers with t_1/2_ ~ 3 h. GFP–Tau^FL-wt^ (blue, t_1/2_ ~ 2 h) and Tau^FL-wt^–GFP (magenta, t_1/2_ ~ 12 h) aggregate with distinct rates and reach higher final levels, consistent with the higher mass of the subunits and variations in elongation. **(b)** Unlabeled Tau^RDΔK^ (green curve) shows aggregation with t_1/2_ ~ 6 h into PHF-like fibers. By contrast, GFP-Tau^RDΔK^ (blue) shows no increase in light scattering, and Tau^RDΔK^–GFP (magenta) reaches only a low level of light scattering saturating at ~ 2 h. Thus in both cases the aggregation of GFP-labeled protein is severely inhibited. **(c-h)** Electron microscopy reveals heterogeneous aggregation forms of Tau + GFP Samples of Tau^FL-wt^, Tau^RDΔK^ with and without GFP aggregated for 24 h at 37 °C were placed on carbon grids and imaged by negative stain electron microscopy. Top row, Tau^FL-wt^ proteins with or without GFP forms typical long filaments (**c**-**e**), whereas repeat domain Tau^RDΔK^ does not (**f**-**h**, bottom row). **(c)** Tau^FL-wt^ aggregates into typical PHF-like twisted filaments (diameter ~ 28 nm). **(d)** GFP-Tau^FL-wt^ forms long filaments with increased diameter (~ 40 nm). **(e)** Tau^FL-wt^-GFP forms long filaments and bundles (~ 28 nm). **(f)** Repeat domain Tau^RDΔK^ without GFP forms filaments of typical PHF-like morphology but smaller diameter (~ 21 nm). **(g)** GFP-Tau^RDΔK^ does not form filaments but only amorphous small aggregates. **(h)** Tau^RDΔK^-GFP forms short filaments and oligomers with the length < 100 nm; average diameter ~ 37 nm

The central question of this paper is to see whether GFP fusion of the Tau repeat domain allows aggregation into bona fide PHF-like filaments. To test this, Tau^FL-wt^ (Fig. 4a) and Tau^RDΔK^ (Fig. 4b) and with and without GFP were incubated with the nucleating reagent heparin 16000 at 37 °C, and aggregation was monitored by UV light scattering at 350 nm. In the case of the repeat domain, the overall extent of aggregation of Tau^RDΔK^ (Fig. 4b, green) was 3-fold higher than that of Tau^RDΔK^-GFP (magenta) whereas GFP-Tau^RDΔK^ (blue) showed almost no response, indicating that GFP attached to either end of the Tau repeat domain was strongly inhibitory, even though it carried the pro-aggregant mutation ΔK280. A different picture emerges from full length Tau: Here the unlabeled and GFP-labeled proteins showed a robust increase of light scattering during assembly (Fig. 4a), with some variation in assembly rates and final levels. Note that GFP increases the Tau subunit mass by ~28kD/48kD ~ 58%, roughly consistent with the increase in scattering.

### GFP fused Tau^RDΔK^ can form amorphous small oligomers and aberrant filaments

The data from light scattering and sedimentation assays (data not shown) were correlated with structural investigations using electron and atomic force microscopy. Tau^FL-wt^ aggregated into long (straight and twisted) fibrils (apparent diameter ~ 28 nm, Fig. 4c).

GFP-Tau^FL-wt^ also formed straight or twisted filaments (Fig. 4d) but appeared somewhat thicker in negative stain (~ 40 nm), consistent with the extra mass of GFP. Tau^FL-wt^-GFP formed long, straight or twisted fibrils with a tendency to coalesce into bundles (Fig. 4e). As a control, the His-tag on Tau^FL-wt^-^His^ had no influence on assembly (similar to Fig. 4c, data not shown).

More pertinent to the present study are the results on the shorter construct Tau^RDΔK^ which readily forms PHF-like fibers with β-structure (Fig. 4f, see [9]). However, in contrast to full-length Tau, filament formation was strongly inhibited by GFP-tags on Tau^RDΔK^. Fusion of GFP to the N-terminus (GFP-Tau^RDΔK^) completely abolished fiber aggregation, and only small amorphous assemblies were generated (Fig. 4g). GFP fusion to Tau^RDΔK^ at C-terminus (Tau^RDΔK^-GFP) made it difficult to purify in E.coli due to protein instability, and hence the protein was expressed in Sf9 cells. This protein formed predominantly oligomers and few elongated particles with thicker diameters, distinct from PHFs (~ 37 nm, Fig. 4h).

### Atomic force microscopy of fibrils assembled from full-length Tau-GFP reveals GFP decoration around the core

**Fig. 5:**
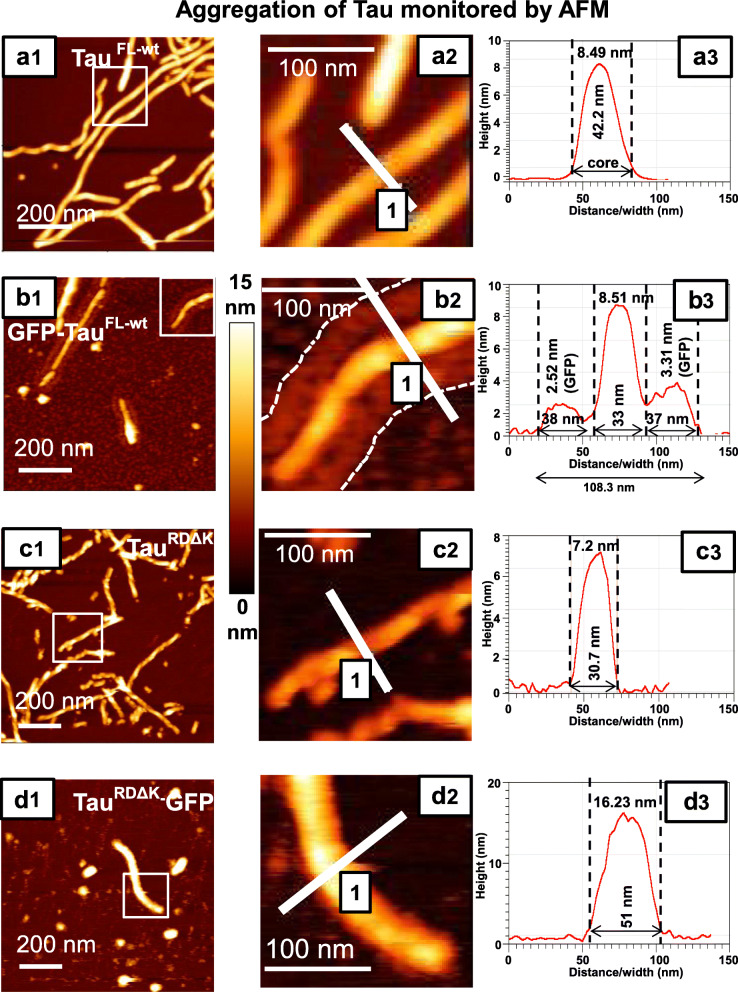
Atomic force microscopy reveals the changes in Tau filament assembly and decoration with GFP. AFM analysis of Tau fibrils formed in the presence of heparin for 24 h at 37 °C was imaged in tapping mode using an MSNL cantilever. Left row, overviews; center row, magnified details; right row, line scans showing distribution of height. Tau^FL-wt^**(a1)** and GFP-Tau^FL-wt^**(b1)** both form long fibrils (straight or twisted), but the height distribution shows characteristic differences because the attachment of GFP generates a halo on both sides of the filament core (compare a3 and b3, core and side bands indicated by dashed lines). The average height (thickness) of the fibril core is similar for Tau^FL-wt^ (7.7 nm ± 0.7 nm; *n* = 36) and GFP-Tau^FL-wt^ (7.0 nm ± 1.2 nm; *n* = 36). However, the overall thickness increases from Tau^FL-wt^ alone (38.1 nm ± 5.45 nm; *n* = 50) to GFP-Tau^FL-wt^ (96.01 nm ± 8.98 nm; *n* = 50). The two side peaks (b3) are ~ 3 nm wide, in good agreement with the shape of GFP. (**c-d**) The repeat domain constructs Tau^RDΔK^ forms well twisted filaments **(c1)** whereas Tau^RDΔK^-GFP forms only few short filaments and more globular shaped aggregates **(d1)**. The enlarged images **(c2, d2)** show that the short filaments of Tau^RDΔK^-GFP are thicker than those of Tau^RDΔK^. (**c3** and **d3)** The widths and heights of Tau^RDΔK^-GFP (width - 42.95 nm ± 8.32 nm; *n* = 50; height- 18.5 nm ± 3.1 nm; *n* = 36) fibrils are larger than those of Tau^RDΔK^ fibrils (width - 28.17 nm ± 3.35 nm; *n* = 50; height - 7.0 nm ± 1.1 nm; *n* = 36). The height scale for a1, b1, c1 and d1 is 0 to 15 nm

As an alternative imaging approach, we used AFM in tapping mode of unstained Tau aggregates. The spatial resolution in the x-y-plane is limited by the size of the tip, but the z-height is recorded accurately via the tip touching the protein surface. Figure 5 shows overview images of Tau filaments (left), magnified views (middle), and plots of the height distribution across filaments (right). Tau^FL-wt^ shows long twisted or straight filaments (Fig. 5a) with heights of ~ 8 nm and apparent widths of ~ 42 nm, enlarged ~ 2-fold by the tip-broadening effect, compared with the actual width 10–25 nm [75]. Filaments of GFP-Tau^FL-wt^ (Fig. 5b) show a similar core (height ~ 7 nm), but in addition a surrounding halo of height ~ 3 nm which matches the size of the GFP moiety. Unlike GFP tagged full length Tau, Tau^RDΔK^-GFP aggregates are mostly small oligomers and some short thick filaments with height of ~ 16 nm (Fig. 5d), about twice the value of unlabeled fibers (Fig. 5c).

Taken together, the kinetic and structural data show that attachment of GFP to Tau^RDΔK^ on either side severely inhibits its aggregation, and the small fraction of fiber-like aggregates is distinct from PHFs.

### Mass-per-length analysis by STEM discriminates PHF-like filaments from aberrant structures

**Fig. 6:**
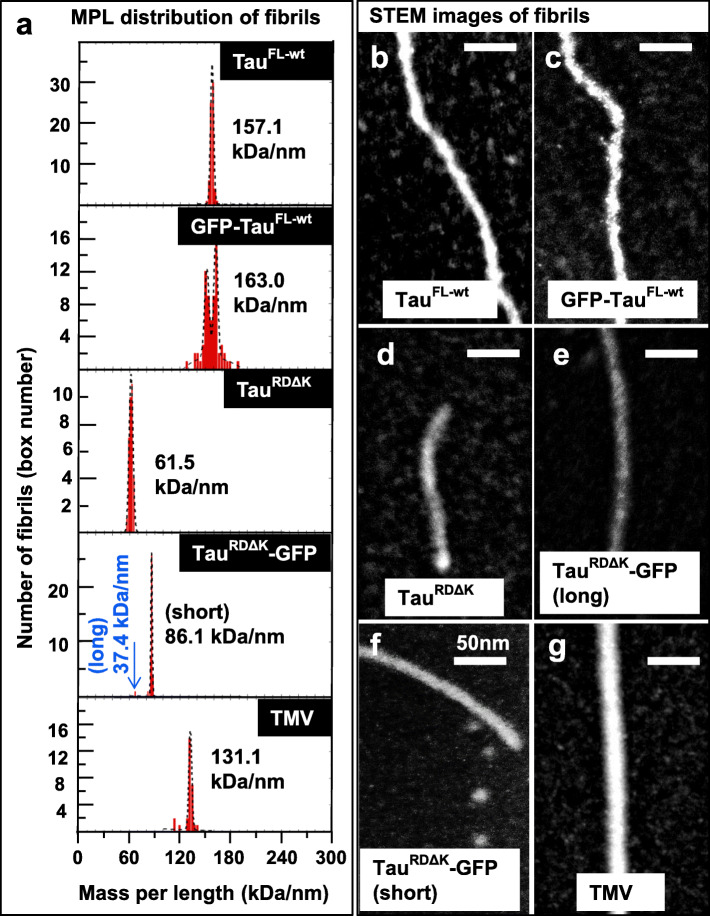
STEM imaging reveals that packing of Tau in assembly conditions is severely altered by GFP tags. **(a)** Mass per length histograms (MPL) of fibrils aggregated for 24 h at 37 °C, with fitted peak values (in kDa/nm) listed for each protein. Examples of STEM images are shown for Tau^FL-wt^**(b)**, GFP-Tau^FL-wt^**(c)**, Tau^RDΔK^**(d)**; Tau^RDΔK^-GFP fibrils (long) **(e)** and Tau^RDΔK^-GFP fibrils (short) **(f)**. MPL measurements were calibrated with tobacco mosaic virus (TMV, shown in the bottom histogram in **a** and in image **g**). The estimated molecules per nm are presented in Table 1. Note that Tau^RDΔK^-GFP fibrils have been observed mostly as short and stubby fibrils, but that the long fibrils show a packing, less dense than the short fibrils

PHFs from Alzheimer brains typically consist of two protofilaments with cross-β structure [22]. Given the axial spacing between β-strands of ~ 0.47 nm, the molecular density of protein subunits would be 1/0.47 = 2.13 units per nm in each protofilaments, or ~ 4.26 molecules per nm in a PHF, irrespective of the molecular weight of the subunits. Thus the mass-per-length (MPL) should be ~ 4.2 times the subunit MW. The MPL value can be determined by STEM which allows one to distinguish PHF-like subunit packing from aberrant structures. Tau filaments with and without GFP were prepared at 37 °C for 24 h for STEM analysis and the MPL data were compared with standard MPL data of tobacco mosaic virus (TMV). The results (Fig. 6 and Table 1) show that fibers assembled from the unlabeled repeat domain Tau^RDΔK^ agree very well with the theoretical expectations (~ 4.4 molecules/nm). Fibers from unlabeled Tau^FL-wt^ have ~ 20% lower values, ~ 3.4 molecules/nm, even though their appearance by EM or AFM is similar to those of the repeat domain. Both observations are in excellent agreement with our earlier study using a different set of instruments and protein preparations [69]. The apparent decrease by ~ 20% of Tau^FL-wt^ can be explained by the fact that the “fuzzy coat” of full-length Tau is spread out around the perimeter so that its contrast is partly buried in the background. On the other hand, in the case of GFP-labeled proteins, the small fraction of fiber-like structures had very different subunit packings, e.g. ~ 0.9 or 2.0 for the “long” and “short” fibrils of Tau^RDΔK^-GFP, and ~ 2.2 for GFP-Tau^FL-wt^ fibrils. These values are clearly incompatible with a PHF-like packing of molecules, yet their GFP moieties are close enough to generate FRET signals. MPL data evidently show that GFP fusion changes the aggregation pattern and packing of Tau molecules in filaments (see Table 1 for MPL values).

**Table 1:** Summary of the effects of Tau + GFP fusion proteins on aggregation, stimulation of microtubule assembly, and packing of subunits in Tau filaments

Construct	Expression (Cell type)	MT assembly	Aggregation (+Heparin)	Mol. weight (kDa)	MPL (kDa/nm)	No.of. molecules /nm
**Tau**^FL-wt^	**E.*coli***	**yes**	**Fibrils**	**45.85**	**157.1±4**	**3.4**
**GFP-Tau**^FL-wt^	**E.*coli***	**yes**	**Fibrils**	**73.76**	**163.0±5**	**2.2**
**Tau**^FL-wt^**-GFP**	**E.*coli***	**yes**	**Fibrils**	**74.54**	**n.d.**	
**Tau**^RDΔK^	**E.*coli***	**no**	**Fibrils**	**13.55**	**61.5±4**	**4.5**
**GFP-Tau**^RDΔK^	**E.*coli***	**no**	**no**	**43.07**	**n.a.**	
**Tau**^RDΔK^**-GFP**	**Sf9 cells**	**no**	**Short fibrils (minor fraction)**	**42.77**	**86.1±5**	**2.0**
			**Long fibrils (minor fraction)**	**42.77**	**37.4±7**	**0.87**
**TMV**					**131.1±1 kDa (standard)**	

## Discussion

The progression of Alzheimer Disease can be subdivided into several stages, as judged by abnormal changes in the neuronal protein Tau (notably hyperphosphorylation and aggregation) which spread in the brain with a predictable spatio-temporal sequence, following axonally connected pathways [13]. This suggests that the signal of toxicity spreads via interconnected neurons and/or cells closely associated with them. Various modes of transmitting a toxic signal between cells can be envisaged [14, 71]. Currently one of the favored mechanisms is based on the “prion-like” spreading of Tau protein, whereby a misfolded and aggregation-prone form of Tau is transferred from a donor to an acceptor cell where it nucleates the conversion of native Tau to a misfolded state and thus causes aggregation [33, 67]. This is consistent with the fact that Tau is a neuron-specific protein, and that only neurons develop the abnormal changes of Tau that show up as neurofibrillary tangles or neuropil threads, consisting of bundles of paired helical filaments (PHF) or straight fibers of Tau (SF) [22]. A variant of the hypothesis is that the transfer of pathogenic Tau may occur not directly from donor to acceptor neuron, but indirectly via microglia and exosomes [6].

In the “prion-like” hypothesis, the concept of “seeding” plays an important role. However, the term has acquired two distinct meanings which can lead to ambiguities. In the strict sense (and by analogy with other cytoskeletal fibers [60]), seeding refers to the nucleated self-assembly of native tau into PHF-like filaments in the acceptor cell onto the template provided by the incoming tau, thereby forming tau polymers with a misfolded conformation (Fig. 1). More generally it denotes the transfer of a pathogenic species (“misfolded” Tau) from donor to acceptor cell, resulting in a pathological response (e.g. FRET, phosphorylation, loss of solubility). The tacit assumption is that some species in this transformation (e.g. Tau polymers or oligomers) is pathogenic for the acceptor cell. A widely-used experimental procedure to test the pathogenic potential of a Tau preparation (e.g. from AD brain or transgenic animals) is based on a reporter cell expressing the Tau repeat domain (~ 13 kDa) fused to XFP [GFP or variants] [34]. Exposing the FRET reporter cell to the extracellular Tau preparation may cause a local accumulation of fluorescence (or FRET, if Tau^RD^ is fused to FRET pairs like CFP/YFP). By analogy with the “prion-like” hypothesis, such inclusion with elevated fluorescence or FRET are interpreted as bona fide PHF-like aggregates, resulting from the internalization of the extracellular Tau and subsequent templated assembly of the Tau^RD^-XFP molecules.

**Fig. 7:**
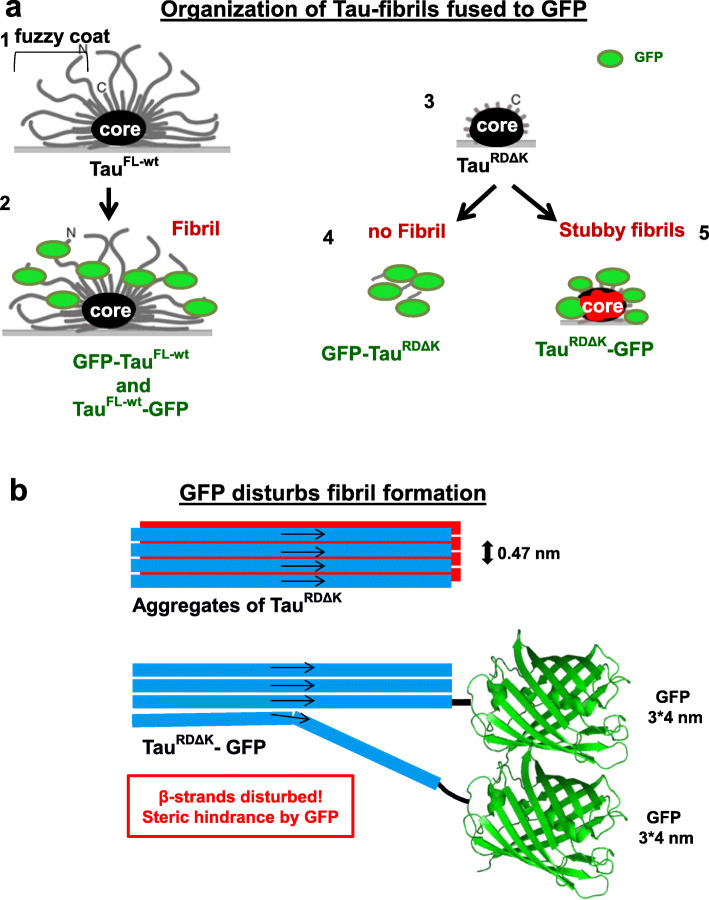
Models of organization of Tau fibrils fused to GFP. **(a)** Models of arrangement of GFP + Tau fusion constructs under aggregating conditions. The diagrams represent filaments lying on a support (grey line), viewed down the filament axis (black oval = core). (1, 2) Tau^FL-wt^ and GFP-Tau^FL-wt^ have a similar packing of their fibril cores (cross-β structure) with attached N- or C-terminal domains (1), plus GFP on either end (2). The PHF-like packing is possible because Tau is disordered and flexible, so that GFP molecules can be accommodated around the perimeter as a fuzzy “halo”. (3) Tau^RDΔK^ can form a PHF-like fibril core, but (4) GFP- Tau^RDΔK^ does not form any fibrils. (5) Tau^RDΔK^-GFP may assemble into short aberrant fibrils, but the arrangement is not compatible with that of AD-like filaments and results in perturbed core (red distorted structure). The GFP creates an outside layer (analogous to the fuzzy coat of PHFs), but the packing is less dense and the elongation is strongly disturbed. **(b)** Diagram of a side view of a PHF-like filament, made up of two parallel β-sheets (red and blue) with antiparallel orientations (arrows). The axial separation of β-strands is 0.47 nm. Size comparison of β-strand and attached GFP. If the GFP were attached sufficiently far away from repeat domain (as in FLTau), the amyloid core could be formed, with GFP molecules accommodated around the perimeter. If the GFP is too close to the β-strand core (as in Tau^RD^) this leads to steric hindrance which causes improper folding and disruption of the amyloid core

We argue that this is an over-interpretation of the fluorescence data on structural grounds. As shown in this paper (Figs. 6 and 7), steric hindrance prevents Tau^RD^-fusion proteins to assemble into PHF-like fibers. In a proper PHF the core has a tightly packed cross-β-structure, with a 0.47 nm distance between adjacent β-strands [22]. However, this is not compatible with the size of an attached GFP molecule (size ~ 3 × 4 nm). Even if a templating Tau assembly were to reach the cytosol of an acceptor cell, it could not combine with the reporter Tau^RD^-GFP fusion molecules to propagate PHF-like structures. This situation is similar to that of other cases where an amyloidogenic protein fused to a compact reporter molecule loses its ability to aggregate into amyloid fibers (e.g. PABPN1-CspB, Aβ-GFP) [15, 56].

How is it then possible to explain local accumulations of GFP-labeled Tau^RD^? One key factor is that the FRET effect extends up to distances of ~ 10 nm, considerably further than the spacing of secondary structures and sizes of the involved protein molecules, and it allows variable relative orientations of molecules. While full-length Tau tends to associate with microtubules, this is not the case for Tau^RD^ which has only a weak affinity for MT (Fig. 3) and instead is rather uniformly distributed in the cytoplasm. However, Tau^RD^ has a natively unfolded structure and can undergo multiple weak interactions with other hydrophilic or charged molecules [53]. Examples are the interactions with ribosomes [44, 58], stress granules [3, 52], transport granules [4, 43, 48]. Moreover, several groups showed recently examples of how the low-complexity composition of Tau or Tau^RD^ enables it to become spontaneously enriched locally in phase-separated membrane-less compartments, particularly in combination with RNA or cytoskeletal proteins [2, 31, 74, 85].

Neurofibrilly changes in Tau were originally described as one of the cellular hallmarks of AD [1]. Their progressive distribution in the brain, correlating with clinical stages [13], and the discovery of disease-causing mutations in Tau [25] led to the concept that pathological changes in Tau protein might be causative via the spreading of Tau between brain cells [17]. This led to a variety of approaches to test the hypothesis, notably cells expressing XFP-labeled full-length Tau or TauRD showing a sensitive FRET reaction [27, 41]. The appearance of FRET was described as a “seeding” reaction, however this term has acquired a dual meaning which can lead to ambiguity: On one hand it refers to nucleated assembly of a Tau filament, on the other it may mean the induction of some pathological property (e.g. accumulation of insoluble material, phosphorylation, changes in fluorescence). Several authors have applied bimolecular complementation assays in cells (e.g. split GFP or split luciferase), which by definition measure the direct binding of two proteins, usually in the ratio of two Tau+one complemented reporter molecule [16, 42, 50, 72, 76]. In these cases one obtains information on dimerization or at best oligomerization, so that conclusions about Tau aggregation cannot be made. An interesting exception is that of [16], who tagged TauRD with a short fragment of GFP and expressed the remainder of GFP separately, leading to a ratio of 1 TauRD+ 1 GFP, the same ratio as we used in vitro. Their conclusion was that GFP prevents PHF assembly in cells, in agreement with our observation. Other authors devised seeding assays with readouts based mainly on the reduction of Tau solubility in cells, e.g. [21, 65]. This increases the range and flexibility of biochemical investigation of triggers (e.g. preparations from AD tissue or transgenic animals) and responses (e.g. abnormal changes in Tau, dependence on mutations), but makes the interpretation in terms of PHF aggregation less specific. Thus, sensor cells are valuable tools in assessing pathogenic properties of extracellular triggers, but there is no need to link this to the templated assembly of Tau filaments. By analogy, in ALS research extracellular stress signals can be tested by their effect on cytoplasmic foci of FUS-GFP, without making assumptions on structures of FUS fibers [46].

Our earlier studies on the problem of somatodendritic Tau “missorting” have shown that changes in Tau distribution can be triggered by extracellular stimuli independently of Tau, for example various stress signals, including oxidative stress or Aβ oligomers, or changes in protein degradation systems [7, 84]. In particular, accumulations of GFP-labeled Tau that mimic seeding can be triggered in neurons by cytokines such as TNFα released from activated microglia [27]. It emphasizes the growing evidence that hallmarks of Tau pathology in neurons can be initiated by inflammatory signals from microglia [37]. This can prompt both the local accumulation, aggregation of assembly-competent Tau as well as other abnormal changes like hyperphosphorylation (e.g. by reducing the activity of phosphatases) or loss of solubility. Noteworthy in this context, “seeding” is observed primarily with heterogeneous Tau preparations that are tissue- or cell-derived, but is inefficient with purified tau samples.

How do these arguments affect approaches to therapy? Earlier attempts to prevent tau pathology were directed at pathological changes of Tau within neurons, assuming they were the carriers of toxicity. Examples are the inhibition of kinases or activation of phosphatases to reduce hyperphosphorylation [47], or inhibitor compounds to block aggregation [59, 77, 78]. Overall these attempts were not successful as treatments. The “prion-like” hypothesis shifted the emphasis to the transfer of tau between cells, which could conceptually be intercepted by Tau-specific antibodies [19, 28, 55, 82]. Despite some encouraging results in transgenic mice, success of this approach is still uncertain and would not be expected if tau protein is not the carrier of pathogenicity, as suggested here.

## Conclusion

Assembly of Tau-GFP is severely inhibited and the aberrant structures formed are incompatible with that of Alzheimer filaments (Fig. 1). Our observations argue against the hypothesis that the propagation of Tau pathology in AD is caused by the prion-like templated aggregation of Tau protein, transmitted via cell-to-cell spreading of Tau. As a consequence, the observed local increase of FRET in recipient cells in Tau-FRET based assays must be caused by alternative processes such as stress granules or liquid liquid phase transition.

## Supplementary information

**Additional file 1 Table S1.** Amino acid sequences of Tau constructs. Amino acid sequences of the Tau constructs used in this study. See also Fig. 2 (a-b). A linker sequence was introduced to separate the GFP from the Tau proteins sequence in the repeat domain Tau constructs. Sequences in green color are GFP protein sequences; blue are linker sequences and red are His tag sequences.

**Additional file 2 Figure S1.** GFP-Tau^FL-wt^ promotes assembly of microtubules in vitro. (a) Microtubules (10 μM) assembled in vitro in presence of untagged Tau^FL-wt^ (5 μM) or (b) GFP-Tau^FL-wt^ (10 μM) were imaged by negative stain electron microscopy. Both Tau constructs efficiently promote the assembly of typical microtubules. (c) Fluorescence microscopy image of microtubules assembled in vitro with GFP-Tau^FL-wt^ reveal the distribution of the GFP tag along the length of microtubules.

## Data Availability

All data generated or analysed during this study are included in this published article [and its supplementary information files].

## Abbreviations

AD: Alzheimer disease
AFM: Atomic force microscopy
CNS: Central nervous system
FRET: Fluorescence resonance energy transfer
GFP: Green fluorescent protein
PHF: Paired helical filament
Tau^FL^: Full-length Tau protein, largest isoform in human CNS (Uniprot P10636-F, hTau40)
Tau^FLΔK^: Full-length Tau protein with pro-aggregant mutation ΔK280
Tau^RD^: Tau repeat domain (amyloidogenic domain)
Tau^RDΔK^: Tau repeat domain with pro-aggregant mutation ΔK280
Sf9: Cell line from *Spodoptera frugiperda*
STEM: Scanning transmission electron microscopy
EM: Electron microscopy
MPL: Mass per length
Wt: Wildtype

## References

[1] Alzheimer A (1907). Über eine eigenartige Erkrankung der Hirnrinde. Allg Z Psychiatr Psychisch Gerichtl Med.

[2] Ambadipudi S, Biernat J, Riedel D, Mandelkow E, Zweckstetter M (2017). Liquid-liquid phase separation of the microtubule-binding repeats of the Alzheimer-related protein tau. Nat Commun.

[3] Apicco DJ, Ash PEA, Maziuk B, Leblang C, Medalla M, Al Abdullatif A, Ferragud A, Botelho E, Ballance HI, Dhawan U, Boudeau S, Cruz AL, Kashy D, Wong A, Goldberg LR, Yazdani N, Zhang C, Ung CY, Tripodis Y, Kanaan NM, Ikezu T, Cottone P, Leszyk J, Li H, Luebke J, Bryant CD, Wolozin B (2018). Reducing the RNA binding protein TIA1 protects against tau-mediated neurodegeneration in vivo. Nat Neurosci.

[4] Aronov S, Aranda G, Behar L, Ginzburg I (2002). Visualization of translated tau protein in the axons of neuronal P19 cells and characterization of tau RNP granules. J Cell Sci.

[5] Arriagada PV, Growdon JH, Hedley-Whyte ET, Hyman BT (1992). Neurofibrillary tangles but not senile plaques parallel duration and severity of Alzheimer's disease. Neurology.

[6] Asai H, Ikezu S, Tsunoda S, Medalla M, Luebke J, Haydar T, Wolozin B, Butovsky O, Kugler S, Ikezu T (2015). Depletion of microglia and inhibition of exosome synthesis halt tau propagation. Nat Neurosci.

[7] Balaji V, Kaniyappan S, Mandelkow E, Wang Y, Mandelkow EM (2018). Pathological missorting of endogenous MAPT/tau in neurons caused by failure of protein degradation systems. Autophagy.

[8] Barghorn S, Biernat J, Mandelkow E (2005). Purification of recombinant tau protein and preparation of Alzheimer-paired helical filaments in vitro. Methods Mol Biol.

[9] Barghorn S, Zheng-Fischhofer Q, Ackmann M, Biernat J, von Bergen M, Mandelkow EM, Mandelkow E (2000). Structure, microtubule interactions, and paired helical filament aggregation by tau mutants of frontotemporal dementias. Biochemistry.

[10] Bieniossek C, Imasaki T, Takagi Y, Berger I (2012). MultiBac: expanding the research toolbox for multiprotein complexes. Trends Biochem Sci.

[11] Biernat J, Gustke N, Drewes G, Mandelkow EM, Mandelkow E (1993). Phosphorylation of Ser262 strongly reduces binding of tau to microtubules: distinction between PHF-like immunoreactivity and microtubule binding. Neuron.

[12] Braak H, Alafuzoff I, Arzberger T, Kretzschmar H, del Tredici K (2006). Staging of Alzheimer disease-associated neurofibrillary pathology using paraffin sections and immunocytochemistry. Acta Neuropathol.

[13] Braak H, Braak E (1991). Neuropathological stageing of Alzheimer-related changes. Acta Neuropathol.

[14] Brundin P, Li JY, Holton JL, Lindvall O, Revesz T (2008). Research in motion: the enigma of Parkinson's disease pathology spread. Nat Rev Neurosci.

[15] Buttstedt A, Winter R, Sackewitz M, Hause G, Schmid FX, Schwarz E (2010). Influence of the stability of a fused protein and its distance to the amyloidogenic segment on fibril formation. PLoS One.

[16] Chun W, Waldo GS, Johnson GV (2007). Split GFP complementation assay: a novel approach to quantitatively measure aggregation of tau in situ: effects of GSK3beta activation and caspase 3 cleavage. J Neurochem.

[17] Clavaguera F, Bolmont T, Crowther RA, Abramowski D, Frank S, Probst A, Fraser G, Stalder AK, Beibel M, Staufenbiel M, Jucker M, Goedert M, Tolnay M (2009). Transmission and spreading of tauopathy in transgenic mouse brain. Nat Cell Biol.

[18] Colby DW, Prusiner SB (2011). De novo generation of prion strains. Nat Rev Microbiol.

[19] Congdon EE, Lin Y, Rajamohamedsait HB, Shamir DB, Krishnaswamy S, Rajamohamedsait WJ, Rasool S, Gonzalez V, Levenga J, Gu J, Hoeffer C, Sigurdsson EM (2016). Affinity of tau antibodies for solubilized pathological tau species but not their immunogen or insoluble tau aggregates predicts in vivo and ex vivo efficacy. Mol Neurodegener.

[20] Devos SL, Miller RL, Schoch KM, Holmes BB, Kebodeaux CS, Wegener AJ, Chen G, Shen T, Tran H, Nichols B, Zanardi TA, Kordasiewicz HB, Swayze EE, Bennett CF, Diamond MI, Miller TM. Tau reduction prevents neuronal loss and reverses pathological tau deposition and seeding in mice with tauopathy. Sci Transl Med. 2017;9:1–10. 10.1126/scitranslmed.aag0481 PMC5792300 28123067

[21] Falcon B, Cavallini A, Angers R, Glover S, Murray TK, Barnham L, Jackson S, O'Neill MJ, Isaacs AM, Hutton ML, Szekeres PG, Goedert M, Bose S (2015). Conformation determines the seeding potencies of native and recombinant tau aggregates. J Biol Chem.

[22] Fitzpatrick AWP, Falcon B, He S, Murzin AG, Murshudov G, Garringer HJ, Crowther RA, Ghetti B, Goedert M, Scheres SHW (2017). Cryo-EM structures of tau filaments from Alzheimer's disease. Nature.

[23] Gaskin F, Cantor CR, Shelanski ML (1975). Biochemical studies on the in vitro assembly and disassembly of microtubules. Ann N Y Acad Sci.

[24] Goedert M, Jakes R, Spillantini MG, Hasegawa M, Smith MJ, Crowther RA (1996). Assembly of microtubule-associated protein tau into Alzheimer-like filaments induced by sulphated glycosaminoglycans. Nature.

[25] Goedert M, Spillantini MG. Tau gene mutations and neurodegeneration. Biochem Soc Symp. 2001:59–71. 10.1042/bss0670059 11447840

[26] Gomez-Ramos A, Diaz-Hernandez M, Rubio A, Miras-Portugal MT, Avila J (2008). Extracellular tau promotes intracellular calcium increase through M1 and M3 muscarinic receptors in neuronal cells. Mol Cell Neurosci.

[27] Gorlovoy P, Larionov S, Pham TT, Neumann H (2009). Accumulation of tau induced in neurites by microglial proinflammatory mediators. FASEB J.

[28] Gu J, Congdon EE, Sigurdsson EM (2013). Two novel tau antibodies targeting the 396/404 region are primarily taken up by neurons and reduce tau protein pathology. J Biol Chem.

[29] Guo JL, Lee VM (2011). Seeding of normal tau by pathological tau conformers drives pathogenesis of Alzheimer-like tangles. J Biol Chem.

[30] Gustke N, Trinczek B, Biernat J, Mandelkow EM, Mandelkow E (1994). Domains of tau protein and interactions with microtubules. Biochemistry.

[31] Hernandez-Vega A, Braun M, Scharrel L, Jahnel M, Wegmann S, Hyman BT, Alberti S, Diez S, Hyman AA (2017). Local nucleation of microtubule bundles through tubulin concentration into a condensed tau phase. Cell Rep.

[32] Holmes BB, Devos SL, Kfoury N, Li M, Jacks R, Yanamandra K, Ouidja MO, Brodsky FM, Marasa J, Bagchi DP, Kotzbauer PT, Miller TM, Papy-Garcia D, Diamond MI (2013). Heparan sulfate proteoglycans mediate internalization and propagation of specific proteopathic seeds. Proc Natl Acad Sci U S A.

[33] Holmes BB, Diamond MI. Cellular models for the study of prions. Cold Spring Harb Perspect Med. 2017;7. 10.1101/cshperspect.a024026 PMC5287063 27815306

[34] Holmes BB, Furman JL, Mahan TE, Yamasaki TR, Mirbaha H, Eades WC, Belaygorod L, Cairns NJ, Holtzman DM, Diamond MI (2014). Proteopathic tau seeding predicts tauopathy in vivo. Proc Natl Acad Sci U S A.

[35] Holtzman DM, Carrillo MC, Hendrix JA, Bain LJ, Catafau AM, Gault LM, Goedert M, Mandelkow E, Mandelkow EM, Miller DS, Ostrowitzki S, Polydoro M, Smith S, Wittmann M, Hutton M (2016). Tau: from research to clinical development. Alzheimers Dement.

[36] Hutton M, Lendon CL, Rizzu P, Baker M, Froelich S, Houlden H, Pickering-Brown S, Chakraverty S, Isaacs A, Grover A, Hackett J, Adamson J, Lincoln S, Dickson D, Davies P, Petersen RC, Stevens M, de Graaff E, Wauters E, van Baren J, Hillebrand M, Joosse M, Kwon JM, Nowotny P, Che LK, Norton J, Morris JC, Reed LA, Trojanowski J, Basun H, Lannfelt L, Neystat M, Fahn S, Dark F, Tannenberg T, Dodd PR, Hayward N, Kwok JB, Schofield PR, Andreadis A, Snowden J, Craufurd D, Neary D, Owen F, Oostra BA, Hardy J, Goate A, van Swieten J, Mann D, Lynch T, Heutink P (1998). Association of missense and 5′-splice-site mutations in tau with the inherited dementia FTDP-17. Nature.

[37] Ising C, Venegas C, Zhang S, Scheiblich H, Schmidt SV, Vieira-Saecker A, Schwartz S, Albasset S, Mcmanus RM, Tejera D, Griep A, Santarelli F, Brosseron F, Opitz S, Stunden J, Merten M, Kayed R, Golenbock DT, Blum D, Latz E, Buee L, Heneka MT (2019). NLRP3 inflammasome activation drives tau pathology. Nature.

[38] Kampers T, Friedhoff P, Biernat J, Mandelkow EM, Mandelkow E (1996). RNA stimulates aggregation of microtubule-associated protein tau into Alzheimer-like paired helical filaments. FEBS Lett.

[39] Kaniyappan S, Chandupatla RR, Mandelkow E (2018). Purification and characterization of low-n tau oligomers. Methods Mol Biol.

[40] Kaniyappan S, Chandupatla RR, Mandelkow EM, Mandelkow E (2017). Extracellular low-n oligomers of tau cause selective synaptotoxicity without affecting cell viability. Alzheimers Dement.

[41] Kfoury N, Holmes BB, Jiang H, Holtzman DM, Diamond MI (2012). Trans-cellular propagation of tau aggregation by fibrillar species. J Biol Chem.

[42] Kim D, Lim S, Haque MM, Ryoo N, Hong HS, Rhim H, Lee DE, Chang YT, Lee JS, Cheong E, Kim DJ, Kim YK (2015). Identification of disulfide cross-linked tau dimer responsible for tau propagation. Sci Rep.

[43] Konzack S, Thies E, Marx A, Mandelkow EM, Mandelkow E (2007). Swimming against the tide: mobility of the microtubule-associated protein tau in neurons. J Neurosci.

[44] Koren SA, Hamm MJ, Meier SE, Weiss BE, Nation GK, Chishti EA, Arango JP, Chen J, Zhu H, Blalock EM, Abisambra JF (2019). Tau drives translational selectivity by interacting with ribosomal proteins. Acta Neuropathol.

[45] Lee G, Cowan N, Kirschner M (1988). The primary structure and heterogeneity of tau protein from mouse brain. Science.

[46] Marrone L, Drexler HCA, Wang J, Tripathi P, Distler T, Heisterkamp P, Anderson EN, Kour S, Moraiti A, Maharana S, Bhatnagar R, Belgard TG, Tripathy V, KALMBACH N, Hosseinzadeh Z, Crippa V, Abo-Rady M, Wegner F, Poletti A, Troost D, Aronica E, Busskamp V, Weis J, Pandey UB, Hyman AA, Alberti S, Goswami A, Sterneckert J (2019). FUS pathology in ALS is linked to alterations in multiple ALS-associated proteins and rescued by drugs stimulating autophagy. Acta Neuropathol.

[47] Medina M. An Overview on the Clinical Development of Tau-Based Therapeutics. Int J Mol Sci. 2018;19(4):1160. 10.3390/ijms19041160 PMC5979300 29641484

[48] Mercken M, Fischer I, Kosik KS, Nixon RA (1995). Three distinct axonal transport rates for tau, tubulin, and other microtubule-associated proteins: evidence for dynamic interactions of tau with microtubules in vivo. J Neurosci.

[49] Michel CH, Kumar S, Pinotsi D, Tunnacliffe A, St George-Hyslop P, Mandelkow E, Mandelkow EM, Kaminski CF, Kaminski Schierle GS (2014). Extracellular monomeric tau protein is sufficient to initiate the spread of tau protein pathology. J Biol Chem.

[50] Mirbaha H, Holmes BB, Sanders DW, Bieschke J, Diamond MI (2015). Tau Trimers are the minimal propagation unit spontaneously internalized to seed intracellular aggregation. J Biol Chem.

[51] Mocanu MM, Nissen A, Eckermann K, Khlistunova I, Biernat J, Drexler D, Petrova O, Schonig K, Bujard H, Mandelkow E, Zhou L, Rune G, Mandelkow EM (2008). The potential for beta-structure in the repeat domain of tau protein determines aggregation, synaptic decay, neuronal loss, and coassembly with endogenous tau in inducible mouse models of tauopathy. J Neurosci.

[52] Moschner K, Sundermann F, Meyer H, da Graca AP, Appel N, Paululat A, Bakota L, Brandt R (2014). RNA protein granules modulate tau isoform expression and induce neuronal sprouting. J Biol Chem.

[53] Mukrasch MD, Bibow S, Korukottu J, Jeganathan S, Biernat J, Griesinger C, Mandelkow E, Zweckstetter M (2009). Structural polymorphism of 441-residue tau at single residue resolution. PLoS Biol.

[54] Nelson PT, Alafuzoff I, Bigio EH, Bouras C, Braak H, Cairns NJ, Castellani RJ, Crain BJ, Davies P, del Tredici K, Duyckaerts C, Frosch MP, Haroutunian V, Hof PR, Hulette CM, Hyman BT, Iwatsubo T, Jellinger KA, Jicha GA, Kovari E, Kukull WA, Leverenz JB, Love S, Mackenzie IR, Mann DM, Masliah E, MCkee AC, Montine TJ, Morris JC, Schneider JA, Sonnen JA, Thal DR, Trojanowski JQ, Troncoso JC, Wisniewski T, Woltjer RL, Beach TG (2012). Correlation of Alzheimer disease neuropathologic changes with cognitive status: a review of the literature. J Neuropathol Exp Neurol.

[55] Novak P, Schmidt R, Kontsekova E, Zilka N, Kovacech B, Skrabana R, Vince-Kazmerova Z, Katina S, Fialova L, Prcina M, Parrak V, dal-Bianco P, Brunner M, Staffen W, Rainer M, Ondrus M, Ropele S, Smisek M, Sivak R, Winblad B, Novak M (2017). Safety and immunogenicity of the tau vaccine AADvac1 in patients with Alzheimer's disease: a randomised, double-blind, placebo-controlled, phase 1 trial. Lancet Neurol.

[56] Ochiishi T, Doi M, Yamasaki K, Hirose K, Kitamura A, Urabe T, Hattori N, Kinjo M, Ebihara T, Shimura H (2016). Development of new fusion proteins for visualizing amyloid-beta oligomers in vivo. Sci Rep.

[57] Pantelic RS, Suk JW, Hao Y, Ruoff RS, Stahlberg H (2011). Oxidative doping renders graphene hydrophilic, facilitating its use as a support in biological TEM. Nano Lett.

[58] Papasozomenos SC, Binder LI (1987). Phosphorylation determines two distinct species of tau in the central nervous system. Cell Motil Cytoskeleton.

[59] Pickhardt M, Neumann T, Schwizer D, Callaway K, Vendruscolo M, Schenk D, ST George-Hyslop P, Mandelkow EM, Dobson CM, McConlogue L, Mandelkow E, Toth G (2015). Identification of small molecule inhibitors of tau aggregation by targeting monomeric tau as a potential therapeutic approach for Tauopathies. Curr Alzheimer Res.

[60] Pollard TD, Borisy GG (2003). Cellular motility driven by assembly and disassembly of actin filaments. Cell.

[61] Pooler AM, Phillips EC, Lau DH, Noble W, Hanger DP (2013). Physiological release of endogenous tau is stimulated by neuronal activity. EMBO Rep.

[62] Prusiner SB (2012). Cell biology. A unifying role for prions in neurodegenerative diseases. Science.

[63] Santacruz K, Lewis J, Spires T, Paulson J, Kotilinek L, Ingelsson M, Guimaraes A, Deture M, Ramsden M, McGowan E, Forster C, Yue M, Orne J, Janus C, Mariash A, Kuskowski M, Hyman B, Hutton M, Ashe KH (2005). Tau suppression in a neurodegenerative mouse model improves memory function. Science.

[64] Schutz AK, Vagt T, Huber M, Ovchinnikova OY, Cadalbert R, Wall J, Guntert P, Bockmann A, Glockshuber R, Meier BH (2015). Atomic-resolution three-dimensional structure of amyloid beta fibrils bearing the Osaka mutation. Angew Chem Int Ed Engl.

[65] Strang KH, Croft CL, Sorrentino ZA, Chakrabarty P, Golde TE, Giasson BI (2018). Distinct differences in prion-like seeding and aggregation between tau protein variants provide mechanistic insights into tauopathies. J Biol Chem.

[66] Tai C, Chang CW, Yu GQ, Lopez I, Yu X, Wang X, Guo W, Mucke L (2020). Tau reduction prevents key features of autism in mouse models. Neuron.

[67] Vaquer-Alicea J, Diamond MI (2019). Propagation of protein aggregation in neurodegenerative diseases. Annu Rev Biochem.

[68] von Bergen M, Barghorn S, Li L, Marx A, Biernat J, Mandelkow EM, Mandelkow E (2001). Mutations of tau protein in frontotemporal dementia promote aggregation of paired helical filaments by enhancing local beta-structure. J Biol Chem.

[69] von Bergen M, Barghorn S, Muller SA, Pickhardt M, Biernat J, Mandelkow EM, Davies P, Aebi U, Mandelkow E (2006). The core of tau-paired helical filaments studied by scanning transmission electron microscopy and limited proteolysis. Biochemistry.

[70] von Bergen M, Friedhoff P, Biernat J, Heberle J, Mandelkow EM, Mandelkow E (2000). Assembly of tau protein into Alzheimer paired helical filaments depends on a local sequence motif ((306) VQIVYK (311)) forming beta structure. Proc Natl Acad Sci U S A.

[71] Walsh DM, Selkoe DJ (2016). A critical appraisal of the pathogenic protein spread hypothesis of neurodegeneration. Nat Rev Neurosci.

[72] Wang Y, Balaji V, Kaniyappan S, Kruger L, Irsen S, Tepper K, Chandupatla R, Maetzler W, Schneider A, Mandelkow E, Mandelkow EM (2017). The release and trans-synaptic transmission of tau via exosomes. Mol Neurodegener.

[73] Wang Y, Mandelkow E (2016). Tau in physiology and pathology. Nat Rev Neurosci.

[74] Wegmann S, Eftekharzadeh B, Tepper K, Zoltowska KM, Bennett RE, Dujardin S, Laskowski PR, Mackenzie D, Kamath T, Commins C, Vanderburg C, Roe AD, Fan Z, Molliex AM, Hernandez-Vega A, Muller D, Hyman AA, Mandelkow E, Taylor JP, Hyman BT. Tau protein liquid-liquid phase separation can initiate tau aggregation. EMBO J. 2018;37(7):e98049. 10.15252/embj.201798049 PMC5881631 29472250

[75] Wegmann S, Jung YJ, Chinnathambi S, Mandelkow EM, Mandelkow E, Muller DJ (2010). Human tau isoforms assemble into ribbon-like fibrils that display polymorphic structure and stability. J Biol Chem.

[76] Wegmann S, Nicholls S, Takeda S, Fan Z, Hyman BT (2016). Formation, release, and internalization of stable tau oligomers in cells. J Neurochem.

[77] Wilcock GK, Gauthier S, Frisoni GB, Jia J, Hardlund JH, Moebius HJ, Bentham P, Kook KA, Schelter BO, Wischik DJ, Davis CS, Staff RT, Vuksanovic V, Ahearn T, Bracoud L, Shamsi K, Marek K, Seibyl J, Riedel G, Storey JMD, Harrington CR, Wischik CM (2018). Potential of low dose Leuco-Methylthioninium Bis (Hydromethanesulphonate) (LMTM) Monotherapy for treatment of mild Alzheimer's disease: cohort analysis as modified primary outcome in a phase III clinical trial. J Alzheimers Dis.

[78] Wischik CM, Harrington CR, Storey JM (2014). Tau-aggregation inhibitor therapy for Alzheimer's disease. Biochem Pharmacol.

[79] Wischik CM, Novak M, Thogersen HC, Edwards PC, Runswick MJ, Jakes R, Walker JE, Milstein C, Roth M, Klug A (1988). Isolation of a fragment of tau derived from the core of the paired helical filament of Alzheimer disease. Proc Natl Acad Sci U S A.

[80] Wu JW, Hussaini SA, Bastille IM, Rodriguez GA, Mrejeru A, Rilett K, Sanders DW, Cook C, Fu H, Boonen RA, Herman M, Nahmani E, Emrani S, Figueroa YH, Diamond MI, Clelland CL, Wray S, Duff KE (2016). Neuronal activity enhances tau propagation and tau pathology in vivo. Nat Neurosci.

[81] Yamada K, Cirrito JR, Stewart FR, Jiang H, Finn MB, Holmes BB, Binder LI, Mandelkow EM, Diamond MI, Lee VM, Holtzman DM (2011). In vivo microdialysis reveals age-dependent decrease of brain interstitial fluid tau levels in P301S human tau transgenic mice. J Neurosci.

[82] Yanamandra K, Kfoury N, Jiang H, Mahan TE, Ma S, Maloney SE, Wozniak DF, Diamond MI, Holtzman DM (2013). Anti-tau antibodies that block tau aggregate seeding in vitro markedly decrease pathology and improve cognition in vivo. Neuron.

[83] Yang F, Moss LG, Phillips GN (1996). The molecular structure of green fluorescent protein. Nat Biotechnol.

[84] Zempel H, Luedtke J, Kumar Y, Biernat J, Dawson H, Mandelkow E, Mandelkow EM (2013). Amyloid-beta oligomers induce synaptic damage via tau-dependent microtubule severing by TTLL6 and spastin. EMBO J.

[85] Zhang X, Lin Y, Eschmann NA, Zhou H, Rauch JN, Hernandez I, Guzman E, Kosik KS, Han S (2017). RNA stores tau reversibly in complex coacervates. PLoS Biol.

