# Supplementary material for: FRET-based Tau seeding assay does not represent prion-like templated assembly of Tau filaments
Source: Mol Neurodegener. 2020 Jul 16;15:39. doi: 10.1186/s13024-020-00389-1 (PMC7364478; doi:10.1186/s13024-020-00389-1)
Supplement: Supplementary file 1 [file 13024_2020_389_MOESM1_ESM.docx]

Table S1

Amino acid sequences of the tau constructs used in this study. See also Figure 1 (A-B). A linker sequence was introduced to separate the GFP from the tau proteins sequence in the repeat domain Tau constructs.

protein sequence	Linker-sequence	GFP sequence	His-Tag

$Tau^{FL\text{-}wt}$ ***(expressed in E.coli)***; 45850 Da

MAEPRQEFEVMEDHAGTYGLGDRKDQGGYTMHQDQEGDTDAGLKESPLQTPTEDGSEEPGSETSDAKSTPTAEDVTAPL
VDEGAPGKQAAAQPHTEIPEGTTAEEAGIGDTPSLEDEAAGHVTQARMVSKSKDGTGSDDKKAKGADGKTKIATPRGAAPP
GQKGQANATRIPAKTPPAPKTPPSSGEPPKSGDRSGYSSPGSPGTPGSRSRTPSLPTPPTREPKKVAVVRTPPKSPSSAKS
RLQTAPVPMPDLKNVKSKIGSTENLKHQPGGGKVQIINKKLDLSNVQSKCGSKDNIKHVPGGGSVQIVYKPVDLSKVTSKCG
SLGNIHHKPGGGQVEVKSEKLDFKDRVQSKIGSLDNITHVPGGGNKKIETHKLTFRENAKAKTDHGAEIVYKSPVVSGDTSP
RHLSNVSSTGSIDMVDSPQLATLADEVSASLAKQGL

$Tau^{FL\text{-}wt}$- 6xHis ***(expressed in E.coli)***; 46673 Da

MAEPRQEFEVMEDHAGTYGLGDRKDQGGYTMHQDQEGDTDAGLKESPLQTPTEDGSEEPGSETSDAKSTPTAEDVTAPL
VDEGAPGKQAAAQPHTEIPEGTTAEEAGIGDTPSLEDEAAGHVTQARMVSKSKDGTGSDDKKAKGADGKTKIATPRGAAPP
GQKGQANATRIPAKTPPAPKTPPSSGEPPKSGDRSGYSSPGSPGTPGSRSRTPSLPTPPTREPKKVAVVRTPPKSPSSAKS
RLQTAPVPMPDLKNVKSKIGSTENLKHQPGGGKVQIINKKLDLSNVQSKCGSKDNIKHVPGGGSVQIVYKPVDLSKVTSKCG
SLGNIHHKPGGGQVEVKSEKLDFKDRVQSKIGSLDNITHVPGGGNKKIETHKLTFRENAKAKTDHGAEIVYKSPVVSGDTSP
RHLSNVSSTGSIDMVDSPQLATLADEVSASLAKQGLHHHHHH

GFP-$Tau^{FL\text{-}wt}$- 6xHis ***(expressed in E.coli)***; 73760 Da

MVSKGEELFTGVVPILVELDGDVNGHKFSVSGEGEGDATYGKLTLKFICTTGKLPVPWPTLVTTLTYGVQCFSRYPDHMKQ
HDFFKSAMPEGYVQERTIFFKDDGNYKTRAEVKFEGDTLVNRIELKGIDFKEDGNILGHKLEYNYNSHNVYIMADKQKNGIKV
NFKIRHNIEDGSVQLADHYQQNTPIGDGPVLLPDNHYLSTQSALSKDPNEKRDHMVLLEFVTAAGITLGMDELYKHMAEPRQ
EFEVMEDHAGTYGLGDRKDQGGYTMHQDQEGDTDAGLKESPLQTPTEDGSEEPGSETSDAKSTPTAEDVTAPLVDEGAP
GKQAAAQPHTEIPEGTTAEEAGIGDTPSLEDEAAGHVTQARMVSKSKDGTGSDDKKAKGADGKTKIATPRGAAPPGQKGQ
ANATRIPAKTPPAPKTPPSSGEPPKSGDRSGYSSPGSPGTPGSRSRTPSLPTPPTREPKKVAVVRTPPKSPSSAKSRLQTA
PVPMPDLKNVKSKIGSTENLKHQPGGGKVQIINKKLDLSNVQSKCGSKDNIKHVPGGGSVQIVYKPVDLSKVTSKCGSLGNI
HHKPGGGQVEVKSEKLDFKDRVQSKIGSLDNITHVPGGGNKKIETHKLTFRENAKAKTDHGAEIVYKSPVVSGDTSPRHLSN
VSSTGSIDMVDSPQLATLADEVSASLAKQGLHHHHHH

$Tau^{FL\text{-}wt}$- GFP-6xHis ***(expressed in E.coli)***; 74540 Da

MAEPRQEFEVMEDHAGTYGLGDRKDQGGYTMHQDQEGDTDAGLKESPLQTPTEDGSEEPGSETSDAKSTPTAEDVTAPL
VDEGAPGKQAAAQPHTEIPEGTTAEEAGIGDTPSLEDEAAGHVTQARMVSKSKDGTGSDDKKAKGADGKTKIATPRGAAPP
GQKGQANATRIPAKTPPAPKTPPSSGEPPKSGDRSGYSSPGSPGTPGSRSRTPSLPTPPTREPKKVAVVRTPPKSPSSAKS
RLQTAPVPMPDLKNVKSKIGSTENLKHQPGGGKVQIINKKLDLSNVQSKCGSKDNIKHVPGGGSVQIVYKPVDLSKVTSKCG
SLGNIHHKPGGGQVEVKSEKLDFKDRVQSKIGSLDNITHVPGGGNKKIETHKLTFRENAKAKTDHGAEIVYKSPVVSGDTSP
RHLSNVSSTGSIDMVDSPQLATLADEVSASLAKQGLGAPGSAGSAAGSGMVSKGEELFTGVVPILVELDGDVNGHKFSVSG
EGEGDATYGKLTLKFICTTGKLPVPWPTLVTTLTYGVQCFSRYPDHMKQHDFFKSAMPEGYVQERTIFFKDDGNYKTRAEV
KFEGDTLVNRIELKGIDFKEDGNILGHKLEYNYNSHNVYIMADKQKNGIKVNFKIRHNIEDGSVQLADHYQQNTPIGDGPVLLP
DNHYLSTQSALSKDPNEKRDHMVLLEFVTAAGITLGMDELYKHHHHHH

$Tau^{RD\Delta K}$ ***(expressed in E.coli)***; 13555 Da

MQTAPVPMPDLKNVKSKIGSTENLKHQPGGGKVQIINKLDLSNVQSKCGSKDNIKHVPGGGSVQIVYKPVDLSKVTSKCGSL
GNIHHKPGGGQVEVKSEKLDFKDRVQSKIGSLDNITHVPGGGNKKIE

GFP- $Tau^{RD\Delta K}$– 6xHis ***(expressed in E.coli)***; 43076 Da

MVSKGEELFTGVVPILVELDGDVNGHKFSVSGEGEGDATYGKLTLKFICTTGKLPVPWPTLVTTLTYGVQCFSRYPDHMKQ
HDFFKSAMPEGYVQERTIFFKDDGNYKTRAEVKFEGDTLVNRIELKGIDFKEDGNILGHKLEYNYNSHNVYIMADKQKNGIKV
NFKIRHNIEDGSVQLADHYQQNTPIGDGPVLLPDNHYLSTQSALSKDPNEKRDHMVLLEFVTAAGITLGMDELYKYSDLELKL
RILQSSMQTAPVPMPDLKNVKSKIGSTENLKHQPGGGKVQIINKLDLSNVQSKCGSKDNIKHVPGGGSVQIVYKPVDLSKVT
SKCGSLGNIHHKPGGGQVEVKSEKLDFKDRVQSKIGSLDNITHVPGGGNKKIEHHHHHH

$Tau^{RD\Delta K}$–GFP-6xHis ***(expressed in Sf9 cells)***; 42778 Da

MQTAPVPMPDLKNVKSKIGSTENLKHQPGGGKVQIINKLDLSNVQSKCGSKDNIKHVPGGGSVQIVYKPVDLSKVTSKCGSL
GNIHHKPGGGQVEVKSEKLDFKDRVQSKIGSLDNITHVPGGGNKKIESTVPRARDPPVATMVSKGEELFTGVVPILVELDGD
VNGHKFSVSGEGEGDATYGKLTLKFICTTGKLPVPWPTLVTTLTYGVQCFSRYPDHMKQHDFFKSAMPEGYVQERTIFFKD
DGNYKTRAEVKFEGDTLVNRIELKGIDFKEDGNILGHKLEYNYNSHNVYIMADKQKNGIKVNFKIRHNIEDGSVQLADHYQQN
TPIGDGPVLLPDNHYLSTQSALSKDPNEKRDHMVLLEFVTAAGITLGMDELYKHHHHHH

GFP-6xHis ***(expressed in E.coli)***; 27762 Da

MVSKGEELFTGVVPILVELDGDVNGHKFSVSGEGEGDATYGKLTLKFICTTGKLPVPWPTLVTTLTYGVQCFSRYPDHMKQ
HDFFKSAMPEGYVQERTIFFKDDGNYKTRAEVKFEGDTLVNRIELKGIDFKEDGNILGHKLEYNYNSHNVYIMADKQKNGIKV
NFKIRHNIEDGSVQLADHYQQNTPIGDGPVLLPDNHYLSTQSALSKDPNEKRDHMVLLEFVTAAGITLGMDELYKHHHHHH
