# Supplementary material for: FRET-based Tau seeding assay does not represent prion-like templated assembly of Tau filaments
Source: Mol Neurodegener. 2020 Jul 16;15:39. doi: 10.1186/s13024-020-00389-1 (PMC7364478; doi:10.1186/s13024-020-00389-1)

Sup.Fig.1

Microtubule assembly

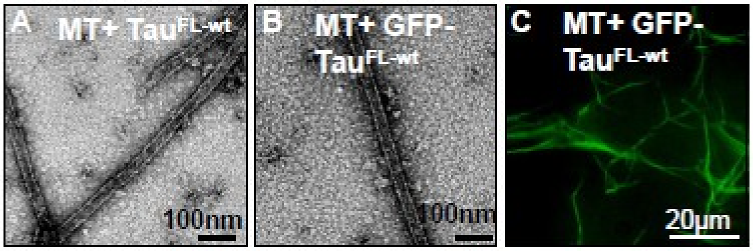

Supplement: Supplementary file 2 [file 13024_2020_389_MOESM2_ESM.docx]
